# Body Mass Index and Mortality among Korean Elderly in Rural Communities: Kangwha Cohort Study

**DOI:** 10.1371/journal.pone.0117731

**Published:** 2015-02-26

**Authors:** Seri Hong, Sang-Wook Yi, Jae Woong Sull, Jae-Seok Hong, Sun Ha Jee, Heechoul Ohrr

**Affiliations:** 1 Department of Preventive Medicine, Graduate School of Public Health, Yonsei University, Seoul, Republic of Korea; 2 Department of Public Health, Yonsei University Graduate School, Seoul, Republic of Korea; 3 Department of Preventive Medicine and Public Health, Catholic Kwandong University College of Medicine, Gangneung, Republic of Korea; 4 Department of Biomedical Laboratory Science, Eulji University College of Health Science, Sungnam, Republic of Korea; 5 Health Insurance Review & Assessment Service, Seoul, Republic of Korea; 6 Department of Epidemiology and Health Promotion, Graduate School of Public Health, Yonsei University, Seoul, Republic of Korea; 7 Institute for Health Promotion, Graduate School of Public Health, Yonsei University, Seoul, Republic of Korea; 8 Department of Preventive Medicine, Yonsei University College of Medicine, Seoul, Republic of Korea; St. Michael's Hospital, CANADA

## Abstract

**Background:**

The relationship between body mass index (BMI) and mortality may differ by ethnicity, but its exact nature remains unclear among Koreans. The study aim was to prospectively examine the association between BMI and mortality in Korean.

**Methods:**

6166 residents (2636 men; 3530 women) of rural communities (Kangwha County, Republic of Korea) aged 55 and above were followed up for deaths from 1985–2008. The multivariable-adjusted hazard ratios were calculated using the Cox proportional hazards model.

**Results:**

During the 23.8 years of follow-up (an average of 12.5 years in men and 15.7 years in women), 2174 men and 2372 women died. Men with BMI of 21.0–27.4 and women with BMI of 20.0–27.4 had a minimal risk for all-cause mortality. A lower BMI as well as a higher BMI increased the hazard ratio of death. For example, multivariable-adjusted hazard ratios associated with BMI below 16, and with BMI of 27.5 and above, were 2.4 (95% CI = 1.6–3.5) and 1.5 (95% CI = 1.1–1.9) respectively, in men, compared to those with BMI of 23.0–24.9. This reverse J-curve association was maintained among never smokers, and among people with no known chronic diseases. Higher BMI increased vascular mortality, while lower BMI increased deaths from vascular diseases, cancers, and, especially, respiratory diseases. Except for cancers, these associations were generally weaker in women than in men.

**Conclusions:**

A reverse J-curve association between BMI and all-cause mortality may exist. BMI of 21–27.4 (rather than the range suggested by WHO of 18.5–23 for Asians) may be considered a normal range with acceptable risk in Koreans aged 55 and above, and may be used as cut points for interventions. More concern should be given to people with BMI above and below a BMI range with acceptable risk. Further studies are needed to determine ethnicity-specific associations.

## Introduction

Obesity defined by body-mass index (BMI) has been a common indicator to determine clinical and public health intervention [1]. However, adiposity among people with the same BMI differs by ethnicity [1–4]. Asian populations are generally thinner but have higher body fat than North American and European populations [1–3]. Therefore, the association of BMI with various diseases in Asians may differ from that in those populations. Although there have been attempts to determine an appropriate BMI range with acceptable risk in Asians [1], the association of BMI with diseases may differ by geographical region even among Asians [5,6], in addition to differing between age groups [7–9].

In contrast to the obesity, being underweight has seldom been recognized as a health risk, despite the fact that several studies have reported this risk [7,10,11]. This is partly due to the low proportion of people with low BMI in North American and European populations [12], and partly to the hypothesis of reverse causation (that is, that low BMI is not a risk but a consequence of underlying conditions leading to poor health outcomes) [8,10]. However, in Asian populations, the proportion of individuals with high and low BMI is reversed from the pattern observed in other populations. Despite the increasing prevalence of obesity in Korea [13], survey results in Korean national representative samples showed that the prevalence of obesity with BMI of 30 or more was just around 3–4%, while the prevalence of underweight with BMI below 18.5 was around 15% in 2010–2012 (authors' analysis of data from the 5^th^ Korean National Health and Nutrition Examination Survey; https://knhanes.cdc.go.kr/knhanes/sub03/sub03_01.jsp). More studies of ethnic and regional groups are needed to better understand the association of low BMI (and high BMI) with the risk of morbidity and mortality from various diseases [6].

The purpose of this study was to prospectively examine the association of BMI with mortality from all causes and specific causes such as cancers (C00-D48), vascular diseases (I00-I99), and respiratory diseases (J00-J99) in Korean elderly people aged 55 or older.

## Methods

### Study population

Kangwha Cohort Study was established in March 1985 [14,15]. Among 9378 residents of Kangwha County in Republic of Korea who were 55 years or older in February 1985, 6372 residents (67.9%) participated in interviews about health behaviors and measurements of blood pressure, height and weight. We excluded participants who were not followed up after the initial survey (n = 39) or those with missing information about their BMI (n = 144) or other covariates (n = 23). Finally, a total of 6166 people (2636 men; 3530 women) were defined as a study population. The study was approved by the Institutional Review Boards of Yonsei University (Approval No. 4-2007-0182). Our results were reported according to the STROBE guidelines [16].

### Baseline Data Collection

Trained investigators interviewed each participant using a structured questionnaire for demographic and health-related factors including smoking, drinking and dietary intake, and measured participants’ height, weight, and blood pressure. Height and weight were determined, with participants wearing light clothing, to the nearest 0.1 cm and 0.1 kg. BMI was calculated as the weight (kg) divided by the square of height (m^2^). More details of the data collection have been described elsewhere [15,17].

### Follow up and outcome assessment

Data on deaths and their causes from 1 January 1992 to 31 December 2008 were confirmed by national death records from the National Statistical Office of Korea. Data on those who died from March 1985 to 31 December 1991 were collected either through calls or visits of trained surveyors twice a year, or from records of burial and death certificates at local administrative branch offices in each study region [15,17]. Follow-up was performed through record linkage at the national level and was complete, except for the case of emigrants. The International Classification of Disease 10^th^ edition (ICD-10) was applied to define the cause of death, which was classified into cardiovascular diseases (I00-I99), cancers (C00-D48), and respiratory diseases (J00-J99) [18].

### Statistical analysis

BMI values were categorized into 7 groups (BMI, kg/m^2^: <16.0, 16.0–18.4, 18.5–20.9, 21.0–22.9, 23.0–24.9, 25.0–27.4, ≥27.5)[1] and into 4 groups (<18.5, 18.5–20.9, 21.0–27.4, ≥27.5).

Cox proportional hazards models were used to calculate hazard ratios after adjustment for covariates. Analyses were performed separately in men and women and were adjusted for the following covariates: age at enrollment (continuous variable), hypertension (hypertension, no hypertension), smoking (never smoked, past smoker, or current smoker), alcohol intake (no drinking, moderate drinking, heavy drinking), fruit and vegetable intake (sufficient, moderate, insufficient), pre-existing chronic diseases (yes, no), health insurance type (Medicaid, National Health Insurance), occupation (agriculture, other), and education level (ever, never).

Tests for linear and quadratic trends were performed to demonstrate the dose-response relationships of BMI to mortality by analyzing BMI categories (1–7, or 1–4) and categories squared as continuous variables. To investigate the potential influence of pre-existing diseases associated with weight loss (or gain) that can contribute to excess risk of death, an analysis was implemented in which those (n = 1010) who died before December 31, 1989 (with less than 4.8 years of follow-up), or those (n = 2955) who had known chronic diseases or cancers were excluded. Additional stratified analysis was performed to examine whether the association between BMI and all-cause mortality differed by smoking status. BMI was further classified into 14 categories (Table A in S1 File) and 3 versions of 4 categories of BMI range (<18.5, 18.5–24.9, 25.0–29.9, ≥30; <18.5, 18.5–22.9, 23.0–24.9, ≥25; <20, 20–21.9, 22–24.9, ≥25) for more detailed information and international comparison. Subgroup analysis and analysis with different BMI categories served as a sensitivity analysis. The p-value was calculated with two-sided tests and a statistical significance level of 0.05 was applied. All statistical analyses were performed using SAS version 9.2 (SAS Inc., Cary, NC, USA).

## Results

The total number of person-years of follow-up was 88,493. During the 23.8 years of follow-up (an average of 12.5 years in men and 15.7 years in women), 4546 participants died (2174 men; 2372 women). At the baseline, the mean (SD) age were 66.3 (7.3) years in men and 66.9 (8.4) years in women. The proportion of underweight with BMI below 18.5 was 9.1% (men, 9.3%; women, 8.9%), while the proportion with BMI of 30 or above was 1.4% (men, 0.4%; women, 2.2%; Table A in S1 File). The majority of men were current smokers and had relatively high education, while women had mostly never smoked and had little education (Table 1). The obese group with BMI of 25 and above tended to be younger, more hypertensive, less likely to be current smokers, fewer farmers, and had more formal education than those with BMI below 25 for both genders (Table B in S1 File).

**Table 1:** Baseline characteristics in Korean elderly (n = 6166).

		Men (n = 2636)	Women (n = 3530)
		n (%)	n (%)
Age (years)	Mean (SD)	66.3 (7.3)	66.9 (8.4)
BMI (kg/m^2^)	<16	27 (1.0)	32 (0.9)
	16–18.4	217 (8.2)	282 (8.0)
	18.5–20.9	854 (32.4)	855 (24.2)
	21–22.9	773 (29.3)	883 (25.0)
	23–24.9	488 (18.5)	714 (20.2)
	25–27.4	204 (7.7)	491 (13.9)
	≥27.5	73 (2.8)	273 (7.7)
Hypertension^a^	No	1,057 (40.1)	1,451 (41.1)
	Yes^a^	1,579 (59.9)	2,079 (58.9)
Smoking	Never	490 (18.6)	2,656 (75.2)
	Past smoker	187 (7.1)	78 (2.2)
	Current smoker	1,959 (74.3)	796 (22.6)
Alcohol intake	None	950 (36.0)	3,177 (90.0)
	Moderate^b^	653 (24.8)	298 (8.4)
	Heavy^b^	1,033 (39.2)	55 (1.6)
Fruit and	Sufficient^c^	87 (3.3)	126 (3.6)
vegetable intake	Moderate^c^	413 (15.7)	498 (14.1)
	Insufficient	2,136 (81.0)	2,906 (82.3)
Known chronic	No	1,454 (55.2)	1,769 (50.1)
disease	Yes	1,182 (44.8)	1,761 (49.9)
Health insurance	Medicaid	146 (5.5)	272 (7.7)
	NHI	2,490 (94.5)	3,258 (92.3)
Occupation	Agriculture	2,255 (85.6)	2,838 (80.4)
	Non-agriculture	381 (14.5)	692 (19.6)
Education level	Ever^d^	1,576 (59.8)	682 (19.3)
	Never	1,060 (40.2)	2,848 (80.7)

BMI, body mass index; NHI, National Health Insurance; SD, standard deviation.

^a^ Hypertension defined as taking hypertension medication regularly or systolic blood pressure ≥140 mm Hg or diastolic blood pressure ≥90 mm Hg.

^b^ Moderate drinking defined as ≤7 drinks/week in women and ≤14 drinks/week in men. Heavy drinking defined as >7 drinks/week in women and >14 drinks/week in men.

^c^ Sufficient intake defined as everyday intake of all components of 1 fruit and 2 vegetable categories; moderate intake defined as nearly-everyday or everyday intake of 1 fruit and at least 1 of 2 vegetable components

^d^ Including elementary/middle/high school, village school (*seodang*), university and college

**Fig 1:**
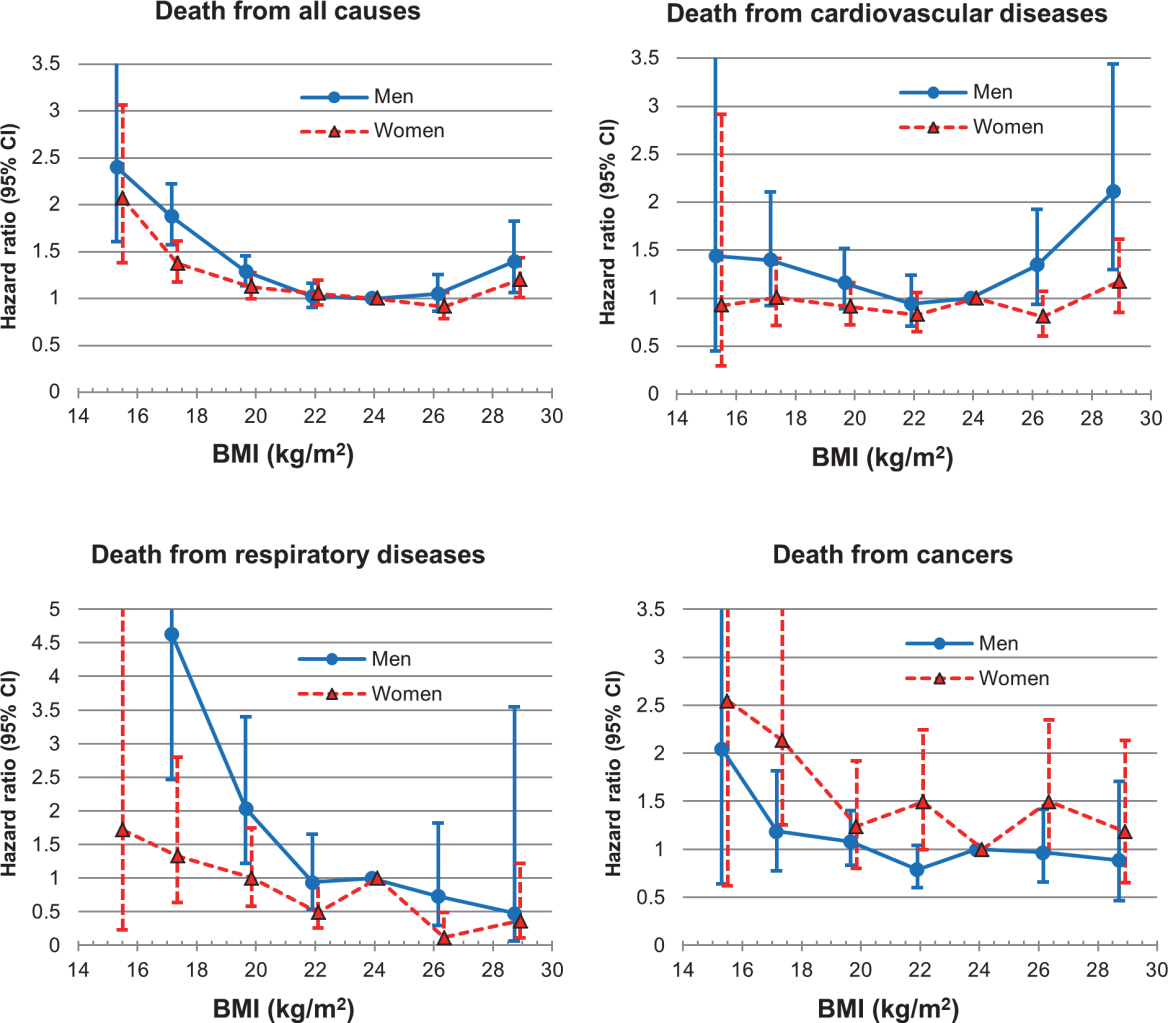
Age-adjusted hazard ratio for mortality from various causes according to gender among the Korean elderly from 1985–2008 by 7 categories of body mass index (BMI) (<16, 16–18.4, 18.5–20.9, 21–22.9, 23–24.9 [Reference], 25–27.4, ≥27.5). The midpoint of each BMI category was used as a representative value for each category, except for both ends of BMI categories, in which the median was used as a representative.

**Fig 2:**
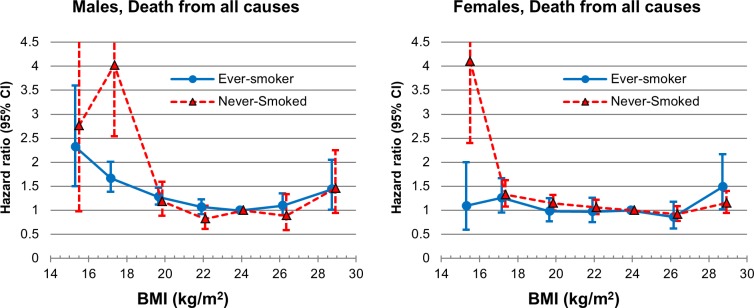
Age-adjusted hazard ratio for all-cause mortality according to smoking status among the Korean elderly from 1985–2008 by 7 categories of body mass index (BMI) (<16, 16–18.4, 18.5–20.9, 21–22.9, 23–24.9 [Reference], 25–27.4, ≥27.5). The midpoint of each BMI category was used as a representative value for each category, except for both ends of BMI categories, in which the median was used as a representative.

Men and women with BMI of 21.0–27.4 (Table 2, Fig. 1), or more specifically, men with BMI of 21.0–27.4 and women with BMI of 20.0–27.4 (Figure A in S1 File), had a minimal age-adjusted risk of death from any cause. In both genders, the lower the BMI, the higher age-adjusted HR, while the highest BMI (≥ 27.5) also had an increased HR compared to those with BMI of 23–24.9. Overall, a reverse J-curve association with a quadratic trend (p<0.001 in both men and women) was found between BMI and all-cause mortality in both genders (Table 2, Fig. 1, Table C in S1 File, Figure B in S1 File). The multivariate-adjusted HRs were similar to age-adjusted HRs in both genders (Table 2). In the stratified analysis by smoking status, the association of the lower end of BMI with all-cause mortality compared to the reference was stronger in those who had never smoked than in past or current smokers (Fig. 2, Figure C in S1 File).

**Table 2:** Adjusted hazard ratios of mortality according to gender by 7 categories of BMI in Korean elderly from 1985–2008.

	Men (n = 2636)						Women (n = 3,530)					
BMI	No. of deaths	(rate)^a^	Age-adjusted		Fully-adjusted^b^		No. of deaths	(rate)^a^	Age-adjusted		Fully-adjusted^b^	
(kg/m2)			HR	(95% CI)	HR	(95% CI)			HR	(95% CI)	HR	(95% CI)
All-cause mortality												
<16	26	(195.1)	2.40	(1.61–3.59)	2.35	(1.57–3.51)	26	(97.4)	2.07	(1.39–3.07)	2.12	(1.42–3.17)
16–18.4	203	(106.6)	1.87	(1.58–2.22)	1.77	(1.49–2.11)	233	(64.4)	1.37	(1.17–1.61)	1.38	(1.17–1.62)
18.5–20.9	737	(74.5)	1.29	(1.14–1.46)	1.26	(1.11–1.43)	627	(50.4)	1.13	(1.00–1.27)	1.14	(1.01–1.29)
21–22.9	603	(57.0)	1.02	(0.90–1.16)	1.01	(0.89–1.15)	571	(39.8)	1.05	(0.93–1.19)	1.04	(0.92–1.18)
23–24.9	382	(55.4)	1.00	Reference	1.00	Reference	454	(39.0)	1.00	Reference	1.00	Reference
25–27.4	162	(57.8)	1.05	(0.87–1.26)	1.06	(0.88–1.27)	279	(32.1)	0.91	(0.79–1.06)	0.89	(0.76–1.03)
≥27.5	61	(70.4)	1.39	(1.06–1.83)	1.47	(1.12–1.92)	182	(41.1)	1.20	(1.01–1.43)	1.14	(0.96–1.35)
p- for linear trend			<0.001		<0.001				<0.001		<0.001	
p- for quadratic trend			<0.001		<0.001				<0.001		<0.001	
Cardiovascular mortality												
<16	3	(22.5)	1.44	(0.45–4.59)	1.55	(0.48–4.95)	3	(11.2)	0.93	(0.30–2.91)	1.17	(0.37–3.72)
16–18.4	32	(16.8)	1.40	(0.93–2.11)	1.41	(0.93–2.13)	45	(12.4)	1.01	(0.72–1.41)	1.08	(0.77–1.52)
18.5–20.9	143	(14.5)	1.16	(0.89–1.52)	1.20	(0.91–1.58)	139	(11.2)	0.91	(0.72–1.16)	0.95	(0.75–1.21)
21–22.9	123	(11.6)	0.94	(0.72–1.25)	0.97	(0.73–1.28)	132	(9.2)	0.83	(0.65–1.06)	0.84	(0.66–1.07)
23–24.9	84	(12.2)	1.00	Reference	1.00	Reference	133	(11.4)	1.00	Reference	1.00	Reference
25–27.4	46	(16.4)	1.35	(0.94–1.93)	1.29	(0.90–1.85)	76	(8.7)	0.81	(0.61–1.07)	0.78	(0.58–1.03)
≥27.5	20	(23.1)	2.11	(1.29–3.44)	1.89	(1.15–3.10)	54	(12.2)	1.17	(0.85–1.61)	1.05	(0.76–1.44)
p- for linear trend			0.536		0.950				0.573		0.598	
p- for quadratic trend			<0.001		<0.001				0.170		0.227	
Respiratory mortality												
<16	0	(0.0)	0		0		1	(3.7)	1.72	(0.23–12.7)	2.1	(0.27–16.2)
16–18.4	19	(10.0)	4.63	(2.47–8.70)	4.04	(2.12–7.68)	10	(2.8)	1.33	(0.64–2.80)	1.39	(0.65–2.96)
18.5–20.9	54	(5.5)	2.04	(1.22–3.40)	1.89	(1.13–3.18)	27	(2.2)	1.01	(0.58–1.75)	1.08	(0.62–1.89)
21–22.9	30	(2.8)	0.94	(0.53–1.65)	0.93	(0.53–1.65)	14	(1.0)	0.49	(0.25–0.95)	0.51	(0.26–0.99)
23–24.9	20	(2.9)	1.00	Reference	1.00	Reference	24	(2.1)	1.00	Reference	1.00	Reference
25–27.4	6	(2.1)	0.73	(0.29–1.82)	0.75	(0.3–1.88)	2	(0.2)	0.12	(0.03–0.49)	0.12	(0.03–0.50)
≥27.5	1	(1.2)	0.48	(0.06–3.55)	0.56	(0.07–4.18)	3	(0.7)	0.37	(0.11–1.21)	0.34	(0.10–1.12)
p- for linear trend			<0.001		<0.001				0.001		<0.001	
p- for quadratic trend			0.093		0.114				0.806		0.768	
Cancer mortality												
<16	3	(22.5)	2.05	(0.64–6.52)	1.79	(0.56–5.71)	2	(7.5)	2.55	(0.61–10.6)	1.98	(0.47–8.36)
16–18.4	28	(14.7)	1.19	(0.64–6.52)	1.12	(0.73–1.71)	22	(6.1)	2.13	(1.25–3.64)	1.97	(1.15–3.39)
18.5–20.9	141	(14.3)	1.08	(0.83–1.40)	1.02	(0.79–1.33)	46	(3.7)	1.24	(0.80–1.92)	1.19	(0.76–1.85)
21–22.9	115	(10.9)	0.79	(0.60–1.04)	0.78	(0.59–1.02)	66	(4.6)	1.50	(1.00–2.24)	1.46	(0.97–2.19)
23–24.9	94	(13.6)	1.00	Reference	1.00	Reference	36	(3.1)	1.00	Reference	1.00	Reference
25–27.4	37	(13.2)	0.97	(0.66–1.41)	0.99	(0.68–1.45)	41	(4.7)	1.50	(0.96–2.35)	1.48	(0.94–2.31)
≥27.5	10	(11.5)	0.89	(0.46–1.70)	1.04	(0.54–2.02)	16	(3.6)	1.18	(0.66–2.13)	1.18	(0.66–2.14)
p- for linear trend			0.226		0.626				0.214		0.374	
p- for quadratic trend			0.084		0.056				0.170		0.258	

BMI, body mass index; CI, confidence interval; HR, hazard ratio

^a^ crude death rate per 1000 person-years

^b^ age at entry (continuous), smoking status (never smoked, past smoker, current smoker), alcohol intake (none, moderate, heavy), fruit and vegetable intake (sufficient, moderate, insufficient), occupation (agriculture, other), education (never, ever), health insurance status (Medicaid, National Health Insurance), known chronic diseases (yes, no), and hypertension (yes, no).

A U-curve association between BMI and cardiovascular mortality was found, in which both the lower and higher end of BMI groups had an increased risk, while decreasing curvilinear-shaped associations were observed between BMI and death from respiratory diseases and cancers, although the decreasing slope was much steeper for death from respiratory diseases than from cancers (Table 2, Fig. 1, Figure B in S1 File)

Except for cancer mortality, the association of both ends of BMI with death from all-cause and specific causes was generally weaker in women than in men (Table 2, Fig. 1, Figures A and B in S1 File). When censoring the first 5-year of follow-up data, or excluding those who had known chronic diseases, the reverse J-curve association between BMI and all-cause mortality remained unchanged (Tables D and E in S1 File). In the meantime, in the analyses by 4 categories of BMI based on international classification by WHO [1] and on Asian classification by WHO Western Pacific Regional Office [19] which is widely used in Korea and Japan, observed associations between BMI and mortality from various causes were somewhat different from those found in the main analyses using 7 categories of BMI (Fig. 2, Tables F and G in S1 File, Figure D in S1 File). The current WHO BMI categories are normal range (18.5–24.9), pre-obese (25.0–29.9), and obesity (≥30) for international classification, while, for the pre-obese and obesity ranges in the Asian population, 23.0–24.9 and ≥25, or 23.0–27.4 and ≥27.5 have been suggested by the WHO Western Pacific Regional Office [19] and WHO expert consultation [1], respectively, due to different adiposity characteristics and potentially different risks associated with BMI in Asians, compared to other populations.

## Discussion

This study found a reverse J-curve association between BMI and all-cause mortality, while various curvilinear types of association were found between BMI and mortality from cancers (decreasing curvilinear shape), cardiovascular diseases (U-curve), and respiratory diseases (decreasing curvilinear shape or negative straight line). The reverse J-curve association with all-cause mortality was maintained in subgroup analyses such as among people who had never smoked, survivors as of January 1990, and people who had no known chronic diseases at enrollment.

### Underweight and Reverse Causation

Our study found that the lower the BMI was, the higher the mortality from all causes. Although several studies have reported this association [7,12,20], it has not been universally accepted [10], mainly due to the hypothesis of reverse causation. Reverse causation suggests that low BMI is not an independent risk factor; rather, that it is a consequence of underlying conditions that cause weight loss and then lead to death. After omitting the first 5 years of follow-up data, or excluding those (n = 2943) with known chronic diseases at enrollment to address the reverse causation [10], the higher all-cause mortality among those with low BMI was generally unchanged. Therefore, there was no clear evidence of reverse causation in the current study, in accordance with previous research, and increased mortality among Kangwha residents with low BMI may not be explained solely by the effect of preexisting conditions [10,21].

### Association between BMI and all-cause mortality

Although, in the ranges of 21.0-27.4 kg/m^2^, the relative risks of all-cause mortality associated with BMI were similar between BMI groups, according to BMI categories for Asians, overweight men with BMI of 23.0–24.9 and women with mild obesity with BMI of 25.0–27.4 were the groups with the lowest adjusted HR in each gender in this study (Table 2). These results are generally consistent with other large cohort studies among Koreans or East Asians [6,22]. These findings that overweight and mild obesity was included at the ranges with a minimal risk, are also in line with a recent systemic review [20], although the reported BMI ranges for overweight and mild obesity were different due to our study using ranges for Asians. In the current study, overall mortality risk started increasing from BMI of 21 or below, even though BMI between 18.5–21.0 is considered a normal BMI [1]. At the higher end of BMI, our results showed that the risk of mortality started increasing from BMI of 27.5 or above [6,22]. Results from our study, as well as from other large cohort studies [6,21,22], suggest that the lower end of the normal BMI range may be revised to at least 21 kg/m^2^ from the previously suggested 18.5, and additionally that instead of 23, around 27.5 may be suitable for the upper end of normal BMI among elderly populations at ages of 55 years and above in Korean or East Asians. However, desirable BMI may be slightly different even among East Asians due to various reasons including diet [23,24].

Meanwhile, when the analysis was restricted to individuals who had never smoked, the risk of overall mortality in those with the lower end of BMI relative to the reference group did not lessen compared to the main analysis in both men and women in the current study, in accordance with previous research among East and South Asians [6]. Therefore, confounding by smoking cannot entirely explain the J-curve association in this study. Hazard modification by gender (namely, a weak association of both ends of the BMI range with all-cause mortality in women compared to men) has also been observed in other studies among Koreans [22].

All-cause mortality reveals the overall effects on incidence and survival across all causes of death. Although high BMI may be a risk factor for several diseases, including ischemic heart diseases and diabetes, the association of high BMI with modestly increased survival across many conditions could cause a net decrease in the all-cause mortality associated with high BMI, especially among the elderly [8,20]. It is undeniable that people with low BMI have an elevated overall mortality [6,22,25]. It has been, however, largely neglected in the medical community in the era of an obesity epidemic [26]. Many studies, including ours, have shown that smoking (residual confounding) and pre-existing illness (reverse causation) cannot entirely explain the association [6,10,22,25]. Nonetheless, there is room for clarification and further research is needed to confirm the causation and the mechanism behind the association of low BMI with all-cause mortality.

### Association between BMI and cause-specific mortality

The risk of deaths from respiratory diseases was nearly monotonically decreased as BMI increased, in accordance with previous research in Koreans [22], East Asians with BMI equal to or below 30 [6], and Europeans [11,25]. The patterns of association between BMI and deaths from cardiovascular diseases and cancers were similar to studies in East Asians [5,6] and Europeans [11,25], while those were somewhat different from a study in Koreans [22] due to the differences in age distribution, birth cohort, and, partly, exclusion criteria between studies [6]. The association between BMI and cancer mortality in the present study was not substantially changed after omitting the first 5 years of death, or excluding those (n = 45) with known history of cancers at enrollment (Table H in S1 File). In the meantime, despite some differences in the strength or pattern of association between BMI and mortality from cause-specific diseases among studies in Koreans, East Asians, Europeans and North Americans, upon closer scrutiny, the reverse J-curve association of BMI with all-cause mortality was relatively similar between studies, including the current study, when the range of BMI (below around 32) was considered [6,22,25], although the ranges of BMI with the lowest risk may be somewhat different to each other.

### Strengths and limitations of the study

The prospective design and nearly complete long-term follow-up using national mortality data are strengths of our study. Another strength is that BMI was calculated based on the measurement of height and weight by a trained investigator, rather than self-reported information [27]. There are also several limitations in our study. First, the follow-up of death records was different in the 1985–1991 and 1992–2008 periods. However, only 32.5% of deaths occurred during 1985–1991, and even when the deaths from the first 5 years were excluded, the results were similar to the main analysis. Second, the validity of the diagnosis on death certificates was not examined separately. However, validity of causes of death was generally considered to be non-differential according to BMI, and the authors do not consider that this substantially overestimates the hazard ratios. Third, since the number of participants and deaths was small due to various subgroup analyses, especially at both ends of the BMI groups, the statistical power of some analyses may have been decreased. Fourth, our study participants were mostly farmers in rural communities, and they were very thin compared to other populations (even an unusually slim European population) [28]. Our study cannot capture the association of the highest BMIs, such as 40 or above (or even 30 or above), with mortality. Additionally, the association of BMI with mortality may differ by ethnicity. Thus, there may be a limitation to the generalizability of our results, and some of them may not be applied to other populations, especially those who are more obese and have a sedentary lifestyle.

### Implications

BMI has been widely used to determine obesity for public health action as well as clinical intervention [1]. Ethnicity-, gender- and age-specific BMI thresholds may be needed to determine parameters of obesity and underweight with increased risk. Among the elderly in Koreans and East Asians, our results suggest that BMI of 21–27.5 may be recommended as a normal range of acceptable risk, rather than the previously recommended BMI range for Asians of 18.5–23 [1]. Further research is needed to determine an appropriate ethnicity-specific threshold of BMI for interventions, and this should focus on morbidity and mortality from all-causes rather than specific diseases closely related with high BMI such as cardiovascular diseases and diabetes, because focusing on those specific diseases may under- or over-estimate the risk related with low and high BMI from many conditions such as respiratory diseases and lung cancer [25].

More appropriate attention and proper care should be given to those with underweight, since underweight may increase deaths from all causes. Even if the reverse causation hypothesis is confirmed to be true in the future, our suggestion remains valid, because in that case, the underweight individuals are suffering from health problems which have been previously unidentified. This takes into consideration of the evidence from previous studies, including the current study, that extensive exclusion of participants with possible known underlying conditions cannot eliminate the association of low BMI with overall mortality.

### Conclusion

This study suggests that a reverse J-curve association between BMI and all-cause mortality exists, and that men with BMI of 21–27.4 and women with BMI of 20.0–27.4 may have an minimal all-cause mortality among Koreans aged 55 and above. This reverse J-curve association may not be explained entirely by pre-existing illness and uncontrolled confounding due to smoking status. Our study shows that BMI of 21–27.5 may be more acceptable as a range of “normal BMI” among Koreans aged 55 and above than BMI of 18.5–23 among Asians, which was previously recommended by WHO. Further research is needed to determine ethnicity-specific ranges of BMI for overweight and underweight with higher risk. We suggest more concern should be given to people with low BMI, because low BMI may increase all-cause mortality, or underweight individuals may have unidentified underlying conditions that could lead to death

## Supporting Information

S1 File Supplementary tables and figures. A PDF file containing further tables and figures described in this article.
(PDF)
