# Supplementary material for: Body Mass Index and Mortality among Korean Elderly in Rural Communities: Kangwha Cohort Study
Source: PLoS One. 2015 Feb 26;10(2):e0117731. doi: 10.1371/journal.pone.0117731 (PMC4342154; doi:10.1371/journal.pone.0117731)
Supplement: S1 File — A PDF file containing further tables and figures described in this article. (PDF) [file pone.0117731.s001.pdf]

Supplementary Tables and Figures

Body Mass Index and Mortality among Korean Elderly in Rural Communities: Kangwha Cohort Study

Seri Hong, Sang-Wook Yi, Jae Woong Sull, Jae-Seok Hong, Sun Ha Jee, Heechoul Ohrr

3 January, 2015

Correspondence:
Sang-Wook Yi, MD, PhD, Department of Preventive Medicine and Public Health, Catholic Kwandong University College of Medicine, Bumil-ro 579, Gangneung, Gangwon-do, 210-701, Republic of Korea; Tel: +82-33-649-7468; Fax +82-33-641-1074-mail: flyhigh@cku.ac.kr

Table A. Number of the participants and deaths (during 1985-2008) by 14 categories of BMI among Korean elderly in Kangwha county

BMI kg/m^2	Men (N=2,636)				Women (N=3,530)				Total (N=6,166)	
	n	(%)	No. of death	(rate)[a]	n	(%)	No. of death	(rate)[a]	n	(%)
<16	27	(1.0)	26	(195.1)	32	(0.9)	26	(97.4)	59	(1.0)
16-17	38	(1.4)	37	(140.7)	56	(1.6)	49	(76.1)	94	(1.5)
17-18.5	179	(6.8)	166	(101.1)	226	(6.4)	184	(61.9)	405	(6.6)
18.5-20	420	(15.9)	371	(82.5)	428	(12.1)	324	(53.8)	848	(13.8)
20-21	434	(16.5)	366	(67.9)	427	(12.1)	303	(47.3)	861	(14.0)
21-22	414	(15.7)	329	(58.9)	400	(11.3)	253	(39.2)	814	(13.2)
22-23	359	(13.6)	274	(54.9)	483	(13.7)	318	(40.2)	842	(13.7)
23-24	331	(12.6)	259	(54.1)	398	(11.3)	249	(38.1)	729	(11.8)
24-25	157	(6.0)	123	(58.3)	316	(9.0)	205	(40.2)	473	(7.7)
25-26	110	(4.2)	87	(57.3)	252	(7.1)	138	(30.1)	362	(5.9)
26-27.5	94	(3.6)	75	(58.3)	239	(6.8)	141	(34.4)	333	(5.4)
27.5-29	50	(1.9)	43	(74.0)	146	(4.1)	98	(41.7)	196	(3.2)
29-30.5	16	(0.6)	13	(66.4)	74	(2.1)	48	(40.0)	90	(1.5)
≥30.5	7	(0.3)	5	(55.4)	53	(1.5)	36	(41.2)	60	(1.0)

BMI, body mass index.

[a] crude death rate per 1000 person-years

Number of participants with BMI of 30 or above is 10 in men, and 78 in women.

Table B. Baseline characteristics of in Korean elderly(n=6,166) by the three BMI subgroups

		Men (N=2,636)				Women(N=3,530)			
	BMI (kg/m^2)	<18.5	18.5-24.9	≥25	p-value	<18.5	18.5-24.9	≥25	p-value
Variable	Classification	n (%)	n (%)	n (%)		n (%)	n (%)	n (%)	
Age (years)	Mean (SD)	68.9 (8.0)	66.1 (7.2)	65.2 (6.4)	<0.001	70.7 (9.0)	67.1 (8.5)	64.8 (7.3)	<0.001
BMI (kg/m2)	<16	27 (11.1)	-	-		32 (10.2)	-	-	
	16-18.4	217 (88.9)	-	-		282 (89.8)	-	-	
	18.5-20.9	-	854 (40.4)	-		-	855 (34.9)	-	
	21-22.9	-	773 (36.6)	-		-	883 (36.0)	-	-
	23-24.9	-	488 (23.1)	-		-	714 (29.1)	-	
	25-27.4	-	-	204 (73.7)		-	-	491 (64.3)	
	≥27.5	-	-	73 (26.4)		-	-	273 (35.7)	
Hypertension[a]	No	116 (47.5)	864 (40.9)	77 (27.8)	<0.001	150 (47.8)	1,036 (42.3)	265 (34.7)	<0.001
	Yes	128 (52.5)	1,251 (59.2)	200 (72.2)		164 (52.2)	1,416 (57.8)	499 (65.3)	
Smoking	Never	29 (11.9)	380 (18.0)	81 (29.2)		171 (54.5)	1,858 (75.8)	627 (82.1)	
	Former smoker	15 (6.2)	147 (7.0)	25 (9.0)	<0.001	6 (1.9)	54 (2.2)	18 (2.4)	<0.001
	Current smoker	200 (82.0)	1,588 (75.1)	171 (61.7)		137 (43.6)	540 (22.0)	119 (15.6)	
Alcohol intake (drinks/wk)	None	103 (42.2)	743 (35.1)	104 (37.6)		282 (89.8)	2,201 (89.8)	694 (90.8)	
	≤ 0-7(women)/14(men)	61 (25.0)	526 (24.9)	66 (23.8)	0.179	27 (8.6)	215 (8.8)	56 (7.3)	0.733
	> 0-7(women)/14(men)	80 (32.8)	846 (40.0)	107 (38.6)		5 (1.6)	36 (1.5)	14 (1.8)	
Fruit and vegetable intake[b]	Sufficient	10 (4.1)	68 (3.2)	9 (3.3)		10 (3.2)	91 (3.7)	25 (3.3)	
	Moderate	27 (11.1)	330 (15.6)	56 (20.2)	0.072	35 (11.2)	348 (14.2)	115 (15.1)	0.495
	Insufficient	207 (84.8)	1,717 (81.2)	212 (76.5)		269 (85.7)	2,013 (82.1)	624 (81.7)	
Known chronic disease	No	125 (51.2)	1,178 (55.7)	151 (54.5)	0.403	169 (53.8)	1,236 (50.4)	364 (47.6)	0.159
	Yes	119 (48.8)	937 (44.3)	126 (45.5)		145 (46.2)	1,216 (49.6)	400 (52.4)	

Health insurance[c]	Medicaid	20 (8.2)	119 (5.6)	7 (2.5)	0.017	32 (10.2)	201 (8.2)	39 (5.1)	0.005
	National Health Insurance	224 (91.8)	1,996 (94.4)	270 (97.5)		282 (89.8)	2,251 (91.8)	725 (94.9)	
Occupation	Agriculture	207 (84.8)	1,823 (86.2)	225 (81.2)	0.082	245 (78.0)	2,015 (82.2)	578 (75.7)	<0.001
	Non-agriculture	37 (15.2)	292 (13.8)	52 (18.8)		69 (22.0)	437 (17.8)	186 (24.4)	
Education level	Ever[d]	140 (57.4)	1,257 (59.4)	179 (64.6)	0.183	40 (12.7)	452 (18.4)	190 (24.9)	<0.001
	Never	104 (42.6)	858 (40.6)	98 (35.4)		274 (87.3)	2,000 (81.6)	574 (75.1)	

[a] Hypertension defined as on regular hypertension medication or systolic blood pressure ≥140 mm Hg or diastolic blood pressure ≥90 mm Hg.
[b] Sufficient intake defined as everyday intake of all components of 1 fruit and 2 vegetable categories; Moderate intake defined as nearly-everyday or everyday intake of 1 fruit and at least 1 of 2 vegetable components;
[c] Vast majority of Koreans are insured by National Health Insurance, while Medicaid contributes to health of citizens with a low economic status
[d] Including elementary/middle/high school, village school(*seodang*), university and college

Table C. Adjusted hazard ratios of mortality according to gender by 4 categories of BMI in Korean elderly during 1985-2008

BMI (kg/m^2)	Men (N=2,636) No. of death (rate)[a]	Men Age-adjusted HR	Men Age-adjusted (95% CI)	Men Fully-adjusted[b] HR	Men Fully-adjusted[b] (95% CI)	Women (N=3,530) No. of death (rate)[a]	Women Age-adjusted HR	Women Age-adjusted (95% CI)	Women Fully-adjusted[b] HR	Women Fully-adjusted[b] (95% CI)
**All-cause mortality**										
<18.5	229 (112.3)	1.88	(1.63-2.18)	1.80	(1.56-2.08)	259 (66.7)	1.42	(1.24-1.62)	1.44	(1.25-1.65)
18.5-20.9	737 (74.5)	1.26	(1.15-1.39)	1.24	(1.13-1.37)	627 (50.4)	1.12	(1.02-1.24)	1.15	(1.04-1.26)
21-27.4	1,147 (56.6)	1.00	Reference	1.00	Reference	1,304 (37.6)	1.00	Reference	1.00	Reference
≥27.5	61 (70.4)	1.37	(1.06-1.77)	1.45	(1.11-1.88)	182 (41.1)	1.20	(1.03-1.40)	1.15	(0.98-1.34)
p- for linear trend		<0.001		<0.001			<0.001		<0.001	
p- for quadratic trend		<0.001		<0.001			<0.001		<0.001	
**Cardiovascular mortality**										
<18.5	35 (17.2)	1.38	(0.96-1.97)	1.39	(0.97-2.00)	48 (12.4)	1.13	(0.84-1.54)	1.23	(0.90-1.68)
18.5-20.9	143 (14.5)	1.14	(0.93-1.40)	1.17	(0.95-1.44)	139 (11.2)	1.03	(0.85-1.26)	1.08	(0.89-1.32)
21-27.4	253 (12.5)	1.00	Reference	1.00	Reference	341 (9.8)	1.00	Reference	1.00	Reference
≥27.5	20 (23.1)	2.07	(1.16-2.93)	1.84	(1.16-2.93)	54 (12.2)	1.33	(1.00-1.77)	1.19	(0.89-1.59)
p- for linear trend		0.436		0.252			0.754		0.517	
p- for quadratic trend		0.008		0.026			0.065		0.138	
**Respiratory mortality**										
<18.5	19 (9.3)	4.76	(2.82-8.05)	4.15	(2.43-7.10)	11 (2.8)	2.36	(1.20-4.62)	2.44	(1.23-4.85)
18.5-20.9	54 (5.5)	2.19	(1.51-3.19)	2.04	(1.39-2.98)	27 (2.2)	1.74	(1.06-2.85)	1.84	(1.12-3.03)

21-27.4	56 (2.8)	1.00	Reference	1.00	Reference	40 (1.2)	1.00	Reference	1.00	Reference
≥27.5	1 (1.2)	0.51	(0.07-3.69)	0.6	(0.08-4.37)	3 (0.7)	0.64	(0.20-2.06)	0.58	(0.18-1.88)
p- for linear trend		<0.001		<0.001			0.001		<0.001	
p- for quadratic trend		0.974		0.929			0.740		0.605	
**Cancer mortality**										
<18.5	31 (15.2)	1.40	(0.96-2.03)	1.32	(0.90-1.92)	24 (6.2)	1.63	(1.05-2.52)	1.50	(0.96-2.35)
18.5-20.9	141 (14.3)	1.22	(0.99-1.50)	1.16	(0.94-1.43)	46 (3.7)	0.93	(0.67-1.30)	0.90	(0.65-1.27)
21-27.4	246 (12.1)	1.00	Reference	1.00	Reference	143 (4.1)	1.00	Reference	1.00	Reference
≥27.5	10 (11.5)	1.00	(0.53-1.88)	1.18	(0.62-2.24)	16 (3.6)	0.89	(0.53-1.49)	0.90	(0.54-1.52)
p- for linear trend		0.024		0.106			0.132		0.260	
p- for quadratic trend		0.856		0.605			0.278		0.325	

BMI, body mass index; CI, confidence interval; HR, hazard ratio

[a] crude death rate per 1000 person-years

[b] age at entry(continuous), smoking status (never, former, current smoker), alcohol intake (none, moderate, heavy), fruit and vegetable intake occupation (agriculture, other), education (never, ever), health insurance status (Medicaid, National Health Insurance), known chronic diseases (yes, no), and hypertension (yes, no).

Table D. Adjusted hazard ratios of mortality according to gender by 7 categories of BMI in Korean elderly during 1990-2008

BMI (kg/m^2)	Men (N=2,069) No. of death (rate)[a]	Men Age-adjusted HR	Men Age-adjusted (95% CI)	Men Fully-adjusted[b] HR	Men Fully-adjusted[b] (95% CI)	Women (N=3,087) No. of death (rate)[a]	Women Age-adjusted HR	Women Age-adjusted (95% CI)	Women Fully-adjusted[b] HR	Women Fully-adjusted[b] (95% CI)
**All-cause mortality**										
<16	11 (223.0)	2.22	(1.21-4.06)	2.12	(1.15-3.88)	7 (42.0)	0.83	(0.39-1.74)	0.83	(0.39-1.76)
16-18.4	130 (125.0)	1.85	(1.50-2.27)	1.72	(1.40-2.11)	182 (77.1)	1.48	(1.24-1.77)	1.48	(1.23-1.77)
18.5-20.9	516 (82.3)	1.18	(1.03-1.36)	1.16	(1.01-1.34)	492 (56.9)	1.13	(0.99-1.29)	1.13	(0.99-1.30)
21-22.9	460 (63.9)	0.95	(0.83-1.1)	0.94	(0.82-1.09)	479 (46.5)	1.11	(0.97-1.27)	1.09	(0.96-1.25)
23-24.9	311 (66.2)	1.00	Reference	1.00	Reference	370 (44.1)	1.00	Reference	1.00	Reference
25-27.4	133 (70.3)	1.05	(0.86-1.29)	1.07	(0.88-1.32)	250 (39.3)	0.99	(0.84-1.16)	0.96	(0.82-1.13)
≥27.5	49 (90.5)	1.45	(1.08-1.97)	1.53	(1.12-2.07)	155 (49.0)	1.26	(1.04-1.51)	1.19	(0.98-1.44)
p- for linear trend		<0.001		0.004			0.023		0.007	
p- for quadratic trend		<0.001		<0.001			0.002		0.005	

BMI, body mass index; CI, confidence interval; HR, hazard ratio

[a] crude death rate per 1000 person-years

[b] age at entry(continuous), smoking status (never, former, current smoker), alcohol intake (none, moderate, heavy), fruit and vegetable intake occupation (agriculture, other), education (never, ever), health insurance status (Medicaid, National Health Insurance), known chronic diseases (yes, no), and hypertension (yes, no).

Table E. Adjusted hazard ratios of mortality after excluding people who had known chronic diseases or cancer, according to gender by 7 categories of BMI in Korean elderly during 1985-2008 (Sensitivity analysis)

BMI (kg/m^2)	Men (N=1,450) No. of death	(rate)[a]	Age-adjusted HR	(95% CI)	Fully-adjusted[b] HR	(95% CI)	Women (N=1,761) No. of death	(rate)[a]	Age-adjusted HR	(95% CI)	Fully-adjusted[b] HR	(95% CI)
**All-cause mortality**												
<16	10	(137.5)	1.81	(0.96-3.42)	1.75	(0.92-3.33)	10	(121.7)	2.86	(1.52-5.40)	3.06	(1.61-5.80)
16-18.4	106	(99.5)	1.69	(1.34-2.13)	1.55	(1.22-1.96)	126	(59.2)	1.40	(1.12-1.75)	1.38	(1.10-1.73)
18.5-20.9	395	(72.8)	1.28	(1.09-1.51)	1.23	(1.04-1.45)	314	(47.3)	1.09	(0.92-1.3)	1.09	(0.91-1.30)
21-22.9	338	(56.5)	1.01	(0.86-1.20)	0.99	(0.83-1.17)	266	(36.6)	1.08	(0.9-1.29)	1.06	(0.88-1.27)
23-24.9	227	(56.7)	1.00	Reference	1.00	Reference	214	(35.8)	1.00	Reference	1.00	Reference
25-27.4	82	(51.7)	0.93	(0.72-1.2)	0.95	(0.74-1.22)	129	(31.1)	0.97	(0.78-1.21)	0.95	(0.76-1.18)
≥27.5	34	(65.1)	1.28	(0.9-1.84)	1.35	(0.94-1.94)	81	(38.9)	1.32	(1.02-1.71)	1.30	(1.00-1.68)
p- for linear trend			<0.001		<0.001				0.075		0.062	
p- for quadratic trend			<0.001		<0.001				<0.001		<0.001	

BMI, body mass index; CI, confidence interval; HR, hazard ratio

[a] crude death rate per 1000 person-years

[b] age at entry(continuous), smoking status (never, former, current smoker), drinking frequency (none, moderate, heavy), fruit and vegetable intake occupation (agriculture, other), education (never, ever), health insurance status (medicaid, national health insurance), known chronic diseases (yes, no), and hypertension (yes, no).

Table F. Adjusted hazard ratios of mortality according to gender by standard 4 categories of BMI in Korean elderly during 1985-2008

	Men (N=2,636)						Women (N=3,530)					
	No. of death	(rate)[a]	Age Adjusted		Fully Adjusted[b]		No. of death	(rate)[a]	Age Adjusted		Fully Adjusted[b]	
			HR	(95%CI)	HR	(95%CI)			HR	(95%CI)	HR	(95%CI)
**All-cause mortality**												
<18.5	229	(112.3)	1.71	(1.49-1.97)	1.64	(1.43-1.89)	259	(66.7)	1.34	(1.17-1.52)	1.34	(1.17-1.53)
18.5-24.9	1,722	(62.9)	1.00	Reference	1.00	Reference	1,652	(43.0)	1.00	Reference	1.00	Reference
25-29.9	215	(60.6)	1.00	(0.87-1.16)	1.04	(0.90-1.20)	409	(34.5)	0.92	(0.83-1.03)	0.89	(0.80-0.99)
≥30	8	(65.0)	1.23	(0.61-2.46)	1.23	(0.61-2.47)	52	(41.0)	1.23	(0.93-1.62)	1.21	(0.91-1.59)
p- for linear trend			<0.001		<0.001				0.003		<0.001	
p- for quadratic trend			<0.001		<0.001				<0.001		<0.001	
**Cardiovascular mortality**												
<18.5	35	(17.2)	1.35	(0.95-1.91)	1.33	(0.94-1.90)	48	(12.4)	1.10	(0.82-1.49)	1.17	(0.86-1.59)
18.5-24.9	350	(12.8)	1.00	Reference	1.00	Reference	404	(10.5)	1.00	Reference	1.00	Reference
25-29.9	64	(18.0)	1.46	(1.12-1.91)	1.35	(1.03-1.77)	114	(9.6)	0.99	(0.80-1.21)	0.90	(0.73-1.11)
≥30	2	(16.2)	1.53	(0.38-6.14)	1.23	(0.31-4.95)	16	(12.6)	1.42	(0.86-2.34)	1.29	(0.78-2.14)
p- for linear trend			0.207		0.435				0.880		0.421	
p- for quadratic trend			0.010		0.032				0.197		0.182	
**Respiratory mortality**												

<18.5	19	(9.3)	3.32	(2.03-5.43)	2.93	(1.78-4.83)	11	(2.8)	1.65	(0.87-3.14)	1.67	(0.87-3.21)
18.5-24.9	104	(3.8)	1.00	Reference	1.00	Reference	65	(1.7)	1.00	Reference	1.00	Reference
25-29.9	6	(1.7)	0.45	(0.2-1.03)	0.50	(0.22-1.14)	3	(0.3)	0.16	(0.05-0.51)	0.15	(0.05-0.48)
≥30	1	(8.1)	2.62	(0.37-18.8)	2.93	(0.4-21.34)	2	(1.6)	1.1	(0.27-4.51)	0.98	(0.24-4.05)
p- for linear trend			<0.001		<0.001				<0.001		<0.001	
p- for quadratic trend			0.055		<0.001				0.900		<0.001	
**Cancer mortality**												
<18.5	31	(15.2)	1.30	(0.90-1.89)	1.25	(0.86-1.81)	24	(6.2)	1.72	(1.11-2.65)	1.60	(1.03-2.48)
18.5-24.9	350	(12.8)	1.00	Reference	1.00	Reference	148	(3.9)	1.00	Reference	1.00	Reference
25-29.9	46	(13.0)	1.02	(0.75-1.38)	1.10	(0.80-1.50)	53	(4.5)	1.14	(0.83-1.56)	1.15	(0.84-1.58)
≥30	1	(8.1)	0.65	(0.09-4.64)	0.80	(0.11-5.70)	4	(3.2)	0.81	(0.30-2.20)	0.83	(0.31-2.26)
p- for linear trend			0.358		0.719				0.336		0.482	
p- for quadratic trend			0.389		0.304				0.119		0.175	

BMI, body mass index; CI, confidence interval; HR, hazard ratio
[a] crude death rate per 1000 person-years
[b] age at entry(continuous), smoking status (never, former, current smoker), alcohol intake (none, moderate, heavy), fruit and vegetable intake occupation (agriculture, other), education (never, ever), health insurance status (Medicaid, National Health Insurance), known chronic diseases (yes, no), and hypertension (yes, no).

Table G. Comparison of adjusted hazard ratios of all-cause mortality according to various categories of BMI by gender in Korean elderly during 1985-2008

	Men (N=2,636)						Women (N=3,530)					
	No. of death	(rate)[a]	Age Adjusted		Fully Adjusted[b]		No. of death	(rate)[a]	Age Adjusted		Fully Adjusted[b]	
			HR	(95%CI)	HR	(95%CI)			HR	(95%CI)	HR	(95%CI)
**Standard categories for international use**												
<18.5	229	(112.3)	1.71	(1.49-1.97)	1.64	(1.43-1.89)	259	(66.7)	1.34	(1.17-1.52)	1.34	(1.17-1.53)
18.5-24.9	1,722	(62.9)	1.00	Reference	1.00	Reference	1,652	(43.0)	1.00	Reference	1.00	Reference
25-29.9	215	(60.6)	1.00	(0.87-1.16)	1.04	(0.90-1.20)	409	(34.5)	0.92	(0.83-1.03)	0.89	(0.80-0.99)
≥30	8	(65.0)	1.23	(0.61-2.46)	1.23	(0.61-2.47)	52	(41.0)	1.23	(0.93-1.62)	1.21	(0.91-1.59)
p- for linear trend			<0.001		<0.001				0.003		<0.001	
p- for quadratic trend			<0.001		<0.001				<0.001		<0.001	
**Suggested categories for Asian population by WHO Western Pacific Regional Office**												
<18.5	229	(112.3)	1.65	(1.44-1.91)	1.60	(1.39-1.85)	259	(66.7)	1.30	(1.14-1.49)	1.31	(1.14-1.5)
18.5-22.9	1340	(65.5)	1.00	Reference	1.00	Reference	1198	(44.7)	1.00	Reference	1.00	Reference
23-24.9	382	(55.4)	0.87	(0.77-0.97)	0.88	(0.79-0.99)	454	(39.0)	0.92	(0.82-1.02)	0.92	(0.82-1.02)
≥25	223	(60.7)	0.98	(0.85-1.12)	1.01	(0.88-1.17)	461	(35.1)	0.93	(0.83-1.03)	0.89	(0.8-0.99)
p- for linear trend			<0.001		<0.001				<0.001		<0.001	
p- for quadratic trend			<0.001		<0.001				0.003		0.010	
**Suggested categories for Korean elderly by the current study**												

<18.5	229	(112.3)	1.88	(1.63-2.18)	1.80	(1.56-2.08)	259	(66.7)	1.42	(1.24-1.62)	1.44	(1.25-1.65)
18.5-20.9	737	(74.5)	1.26	(1.15-1.39)	1.24	(1.13-1.37)	627	(50.4)	1.12	(1.02-1.24)	1.15	(1.04-1.26)
21-27.4	1,147	(56.6)	1.00	Reference	1.00	Reference	1,304	(37.6)	1.00	Reference	1.00	Reference
≥27.5	61	(70.4)	1.37	(1.06-1.77)	1.45	(1.11-1.88)	182	(41.1)	1.20	(1.03-1.40)	1.15	(0.98-1.34)
p- for linear trend			<0.001		<0.001				<0.001		<0.001	
p- for quadratic trend			<0.001		<0.001				<0.001		<0.001	
**Categories using BMI of 20-21.9 as reference**												
<20	600	(91.8)	1.39	(1.25-1.55)	1.38	(1.23-1.54)	583	(58.9)	1.23	(1.09-1.38)	1.23	(1.1-1.39)
20-21.9	695	(63.3)	1.00	Reference	1.00	Reference	556	(43.2)	1.00	Reference	1.00	Reference
22-24.9	656	(55.2)	0.89	(0.8-0.99)	0.90	(0.81-1.01)	772	(39.5)	1.00	(0.89-1.11)	0.99	(0.88-1.1)
≥25	223	(60.7)	1.00	(0.86-1.17)	1.05	(0.9-1.22)	461	(35.1)	0.97	(0.86-1.1)	0.93	(0.82-1.06)
p- for linear trend			<0.001		<0.001				<0.001		<0.001	
p- for quadratic trend			<0.001		<0.001				0.037		0.084	

BMI, body mass index; CI, confidence interval; HR, hazard ratio

[a] crude death rate per 1000 person-years

[b] age at entry(continuous), smoking status (never, former, current smoker), alcohol intake (none, moderate, heavy), fruit and vegetable intake occupation (agriculture, other), education (never, ever), health insurance status (Medicaid, National Health Insurance), known chronic diseases (yes, no), and hypertension (yes, no).

Table H. Adjusted hazard ratios of cancer mortality according to gender by 7 categories of BMI in Korean elderly without known history of cancer (Sensitivity analysis).

	Men						Women					
	No. of death	(rate)[a]	Age Adjusted		Fully Adjusted[a]		No. of death	(rate)[a]	Age Adjusted		Fully Adjusted[a]	
			HR	(95%CI)	HR				HR	(95%CI)	HR	(95%CI)
**Analysis among participants (2,617 men; 3,504 women) during 1985-2008**												
<16	3	(22.5)	2.1	(0.66-6.70)	1.85	(0.58-5.93)	0	(0.0)	0	-	0	-
16-18.4	25	(13.2)	1.07	(0.69-1.67)	1.01	(0.64-1.57)	22	(6.1)	2.18	(1.27-3.72)	2.01	(1.17-3.47)
18.5-20.9	135	(13.7)	1.04	(0.80-1.35)	0.98	(0.75-1.29)	45	(3.6)	1.24	(0.80-1.94)	1.19	(0.76-1.87)
21-22.9	113	(10.7)	0.77	(0.59-1.02)	0.76	(0.58-1.00)	65	(4.6)	1.51	(1.00-2.28)	1.48	(0.98-2.23)
23-24.9	94	(13.7)	1.00	Reference	1.00	Reference	35	(3.0)	1.00	Reference	1.00	Reference
25-27.4	37	(13.2)	0.96	(0.66-1.41)	0.99	(0.68-1.45)	41	(4.7)	1.54	(0.98-2.42)	1.52	(0.96-2.38)
≥27.5	10	(11.5)	0.88	(0.46-1.70)	1.04	(0.54-2.02)	15	(3.4)	1.15	(0.63-2.10)	1.15	(0.63-2.11)
p- for linear trend			0.451		0.971				0.329		0.524	
p- for quadratic trend			0.124		0.081				0.483		0.618	
**Analysis among survivors as of January 1990 (2,060 men; 3,067 women) (censoring the first 4.8 years of follow-up data) during 1990-2008**												
<16	2	(40.5)	2.98	(0.72-12.3)	2.66	(0.64-11.0)	0	(0.0)	0	-	0	-
16-18.4	15	(14.4)	0.92	(0.53-1.60)	0.85	(0.49-1.49)	16	(6.8)	2.00	(1.09-3.68)	1.77	(0.95-3.29)

18.5-20.9	90	(14.4)	0.88	(0.65-1.19)	0.83	(0.61-1.12)	35	(4.1)	1.17	(0.71-1.90)	1.08	(0.66-1.77)
21-22.9	91	(12.6)	0.75	(0.55-1.01)	0.73	(0.54-0.99)	56	(5.5)	1.53	(0.98-2.38)	1.47	(0.94-2.29)
23-24.9	78	(16.7)	1.00	Reference	1.00	Reference	30	(3.6)	1.00	Reference	1.00	Reference
25-27.4	32	(16.9)	1.01	(0.67-1.52)	1.05	(0.69-1.58)	35	(5.5)	1.51	(0.93-2.47)	1.48	(0.91-2.42)
≥27.5	7	(12.9)	0.80	(0.37-1.72)	0.98	(0.45-2.14)	11	(3.5)	0.99	(0.49-1.97)	1.00	(0.50-2.00)
p- for linear trend			0.617		0.216				0.414		0.750	
p- for quadratic trend			0.312		0.205				0.933		0.867	

BMI, body mass index; CI, confidence interval; HR, hazard ratio

[a] crude death rate per 1000 person-years

[b] age at entry(continuous), smoking status (never, former, current smoker), alcohol intake (none, moderate, heavy), fruit and vegetable intake occupation (agriculture, other), education (never, ever), health insurance status (Medicaid, National Health Insurance), known chronic diseases (yes, no), and hypertension (yes, no).

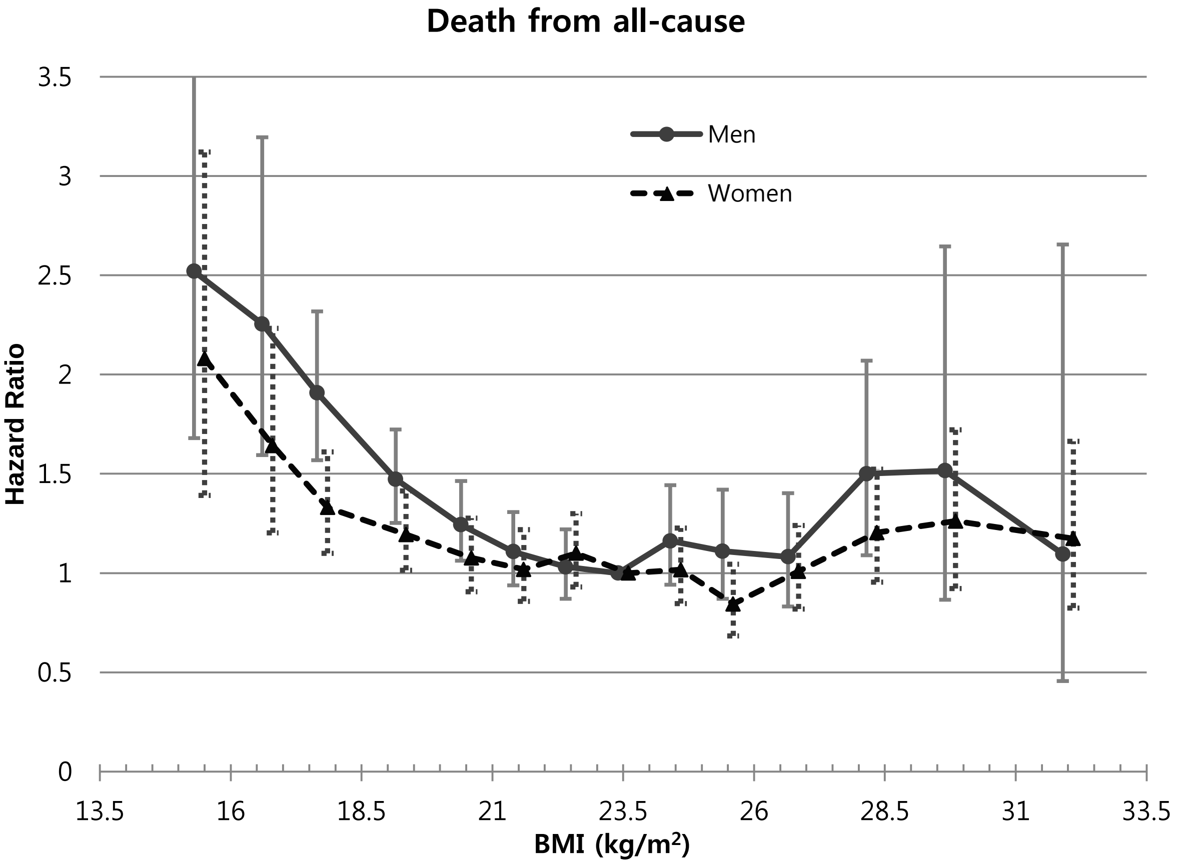

Figure A. Age-adjusted hazard ratio for all-cause mortality according to gender among the Korean elderly during 1985-2008 by 14 categories of body mass index (BMI) (<16, 16-16.9, 17-18.4, 18.5-19.9, 20-20.9, 21-21.9, 22-22.9, 23-23.9 [Reference], 24-24.9, 25-25.9, 26-27.4, 27.5-28.9, 29-30.4, ≥30.5).

The midpoint of each BMI category was used as a representative values of each category, except for the both ends of BMI categories in which median was used as a representative.

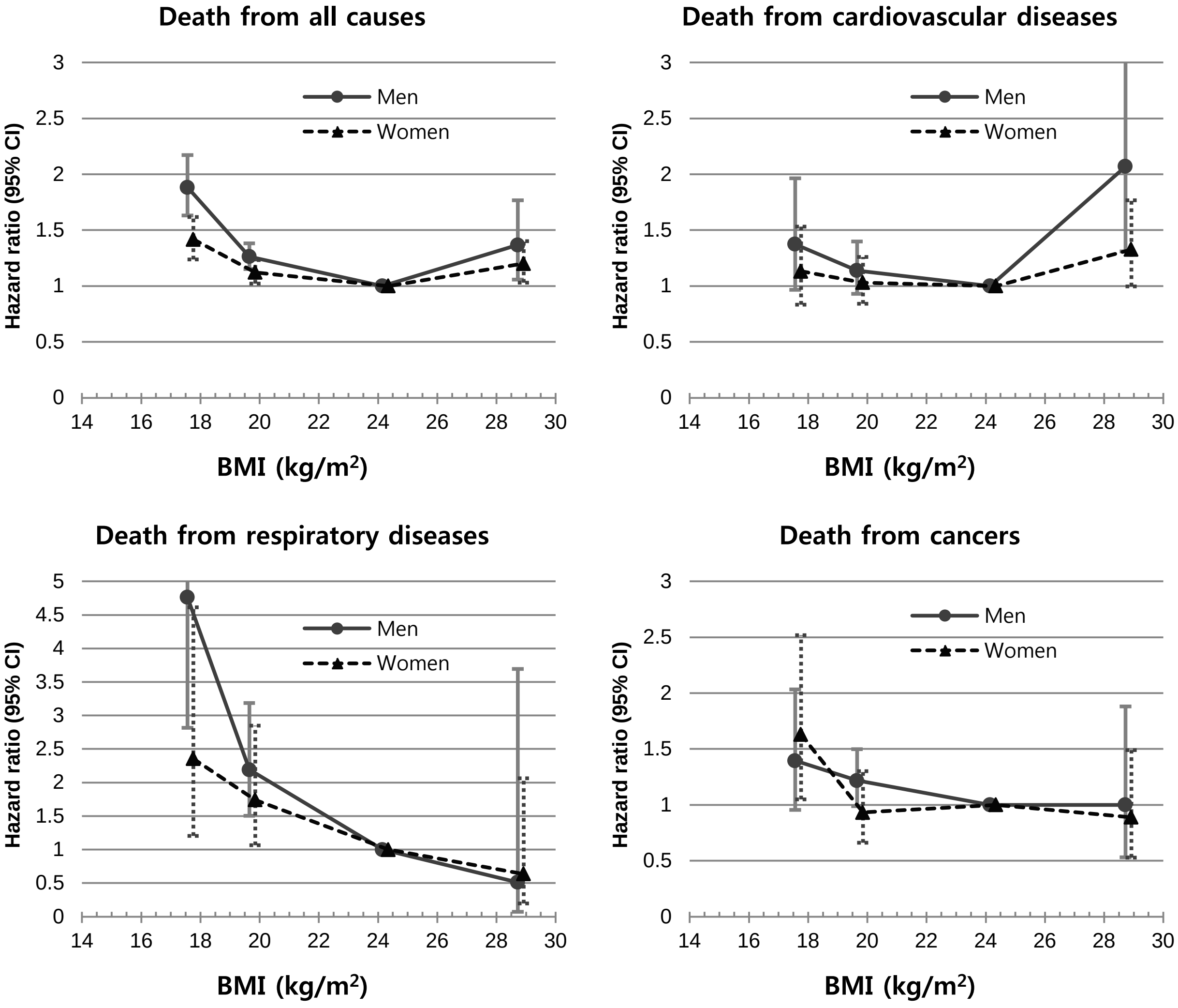

Figure B. Age-adjusted hazard ratio for mortality from various causes according to gender among the Korean elderly during 1985-2008 by 4 categories of body mass index (BMI) (<18.5, 18.5-20.9, 21-27.4 [Reference], ≥27.5). The midpoint of each BMI category was used as a representative value for each category, except for both ends of BMI categories in which the median was used as a representative.

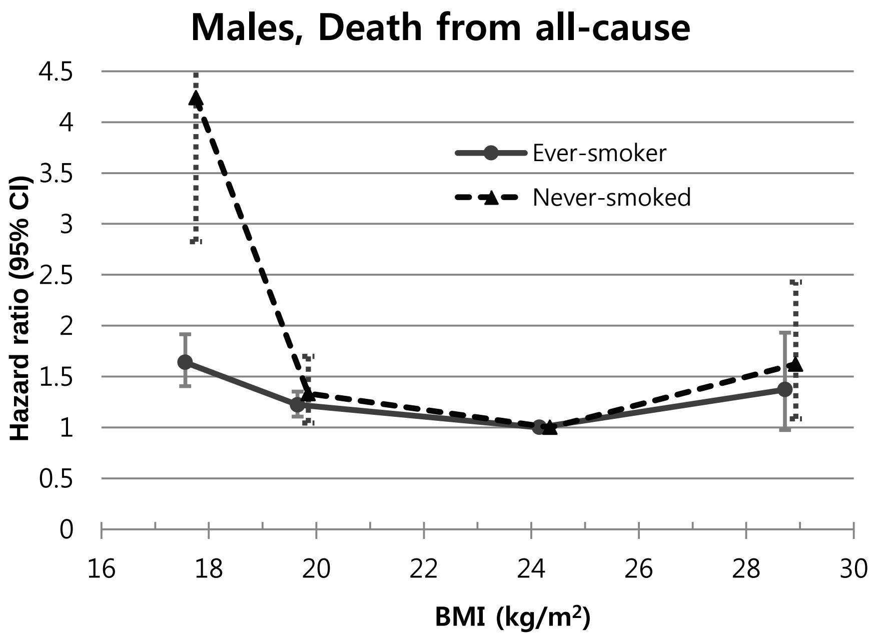

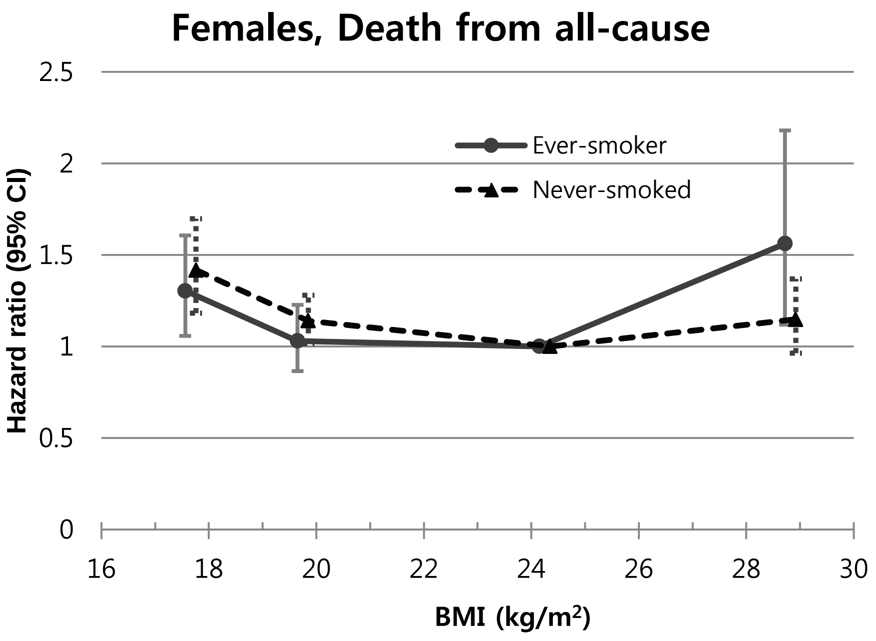

Figure C. Age-adjusted hazard ratio for all-cause mortality according to smoking status among the Korean elderly during 1985-2008 by 4 categories of body mass index (BMI) (<18.5, 18.5-20.9, 21-27.4 [Reference], ≥27.5). The midpoint of each BMI category was used as a representative value for each category, except for both ends of BMI categories in which the median was used as a representative.

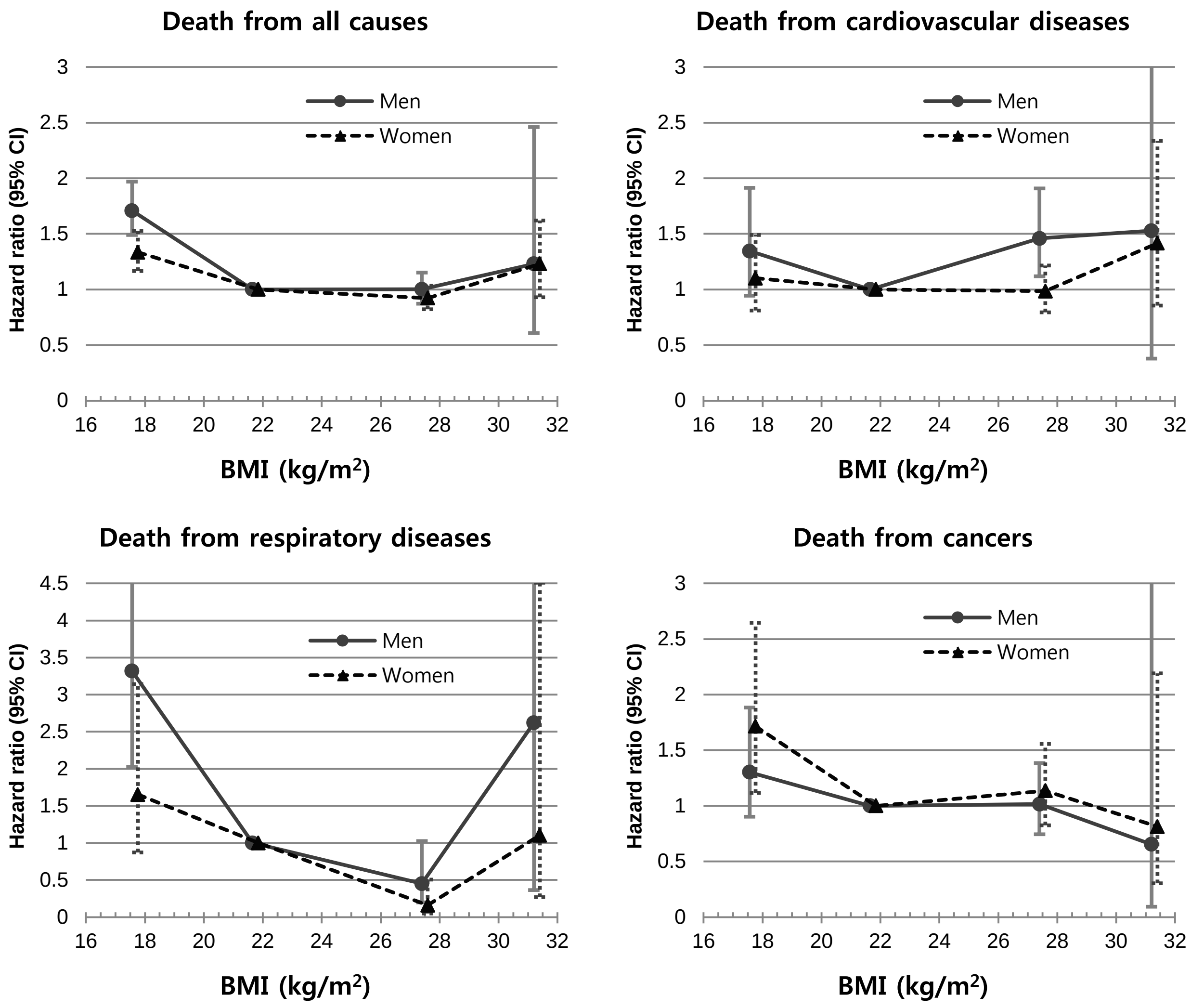

Figure D. Age-adjusted hazard ratio for mortality from various causes according to gender among the Korean elderly during 1985-2008 by 4 categories of body mass index (BMI) based on international classification by WHO (BMI: <18.5, 18.5-24.9 [Reference], 25-29.9, ≥30). The midpoint of each BMI category was used as a representative value for each category, except for both ends of BMI categories in which the median was used as a representative.
